# Enabling good transition processes from child to adult medical care: a study protocol

**DOI:** 10.3389/frhs.2025.1520013

**Published:** 2025-02-27

**Authors:** Camilla Ida Ravnbøl, Laura Altweck, Silke Schmidt, Lene Bistrup, Stefan Borgwardt, Sidse Marie Arnfred, Pia Jeppesen, Philipp von Bismarck, Julie Bork Nellegaard, Alexander Prehn-Kristensen, Ada Colic

**Affiliations:** ^1^ Department of People and Technology, Roskilde University, Roskilde, Denmark; ^2^ Research Department, Zealand University Hospital, Køge, Denmark; ^3^ Department Health and Prevention, Institute of Psychology, University of Greifswald, Greifswald, Germany; ^4^ German Center for Child and Adolescent Health (DZKJ), Partner Site Greifswald/Rostock, Greifswald, Germany; ^5^ Department of Children and Youth, Slagelse, NSR Hospital, Slagelse, Denmark; ^6^ Department of Psychiatry and Psychotherapy, University Medical Center Schleswig-Holstein, Lübeck, Germany; ^7^ Psychiatric Research Unit, Psychiatry Region Zealand, Slagelse, Denmark; ^8^ Department of Child and Adolescent Psychiatry, Copenhagen University Hospital, Psychiatry Region Zealand, Roskilde, Denmark; ^9^ Clinic for Child and Adolescent Medicine, Universitätsklinikum Schleswig-Holstein, Kiel, Germany; ^10^ Trifork Public A/S, Arhus, Denmark; ^11^ Institute for Child and Adolescent Psychiatry, Center for Integrative Psychiatry, Universitätsklinikum Schleswig-Holstein, Kiel, Germany; ^12^ Faculty of Psychology and Human Movement Science, Institute of Psychology, University of Hamburg, Hamburg, Germany; ^13^ Department of Rheumatology, Zealand University Hospital, Køge, Denmark

**Keywords:** transition, child and adolescent health care, adult health care, rheumatology, mental health, mixed-methods, qualitative research

## Abstract

Hundreds of patients each year transfer from child to adult medical care when they become adults. The transfer in health care comes with a risk of interrupted treatment or a failure to follow treatment properly, which can have serious consequences for the physical and mental health and well-being of the young person, and for their future ability to engage in education, work or social life. The Child to Adult Transition project (CAT) is a cross-country and inter-disciplinary innovation and research project that aims to address this pertinent topic. CAT focuses on young people in rheumatology and mental health care in Denmark and Germany and develops transition programmes to support young persons and their parents in the transfer from child to adult medical care, while exploring how young people experience and reflect on this transition and their experiences of the CAT programs. The CAT study has a longitudinal, mixed-methods study design, surveying young patients (age 15–25 years), their parents/guardians, and health-care professionals via interviews (individual or group), field observations, and/or online surveys. At baseline, interviews will be conducted with 24–68 adolescents and young adults, 24–68 parents/guardians, and 24–68 health-care professionals in both countries and across disciplines. 13–14 observations will be made in three settings and, at baseline, 400 adolescents and young adults will receive the survey. Interviews and surveys will be repeated after six and 12 months. The study will focus on topics such as everyday life as a young patient, transition experiences, somatic, and mental health, and quality of life. The CAT project period runs from January 2023 to December 2025. Recruitment to the CAT study is ongoing and all ethical approval have been obtained from the different departmental sites and ethical committees. The project combines different medical disciplines (child, adolescent and adult rheumatology and mental health), academic disciplines (medicine, anthropology and psychology) as well as countries (Germany, Denmark). It also combines person-groups (young persons, parents, professionals) and methods (interviews, observations, surveys). This approach provides new perspectives on the medical, psychological and anthropological aspects of the complex nature of the medical transfer. The findings will feed into the guidelines on transitional care, can also be used in other medical disciplines, and can be prepared as popular publications and other media enabling a broader audience to be reached.

The study protocol is registered on the Open Science Framework: https://osf.io/vdy9p

## 1 Introduction

Becoming an adult comes in a period of a young persons' life where there are a multitude of “firsts', including one's first job, first relationship, or living alone for the first time. On top of this, adolescents and young adults with a serious or chronic illness or diagnosis experience another crucial transition, namely transferring from child and adolescent health-care services (CAHS) to adult health-care services (AHS), which takes place around the age of 18 years (1, 2). At this point, young patients are expected to take greater responsibility for managing their own treatment, which can be overwhelming for both the young patient and their parents or guardians, who cannot provide the same level of support as before (3–6). Studies show that abrupt transfers between medical care departments in the transition process can lead to interruptions in medical treatment and cause serious complications, even early death (3, 7–9). Hence, there is a need for closer cooperation between CAHS and AHS departments and for more coherent approaches in supporting young persons in the transition process.

The project “Child to Adult Transitions: Enabling Good Transition Processes from Child to Adult Medical Care (CAT)” aims to support young patients' transfers from CAHS to AHS within the medical disciplines of rheumatology and mental health care. It covers partners in the northern regions of Germany and the south and southeast regions of Denmark. The overall goal is to improve young patients' transfers from CAHS to AHS by developing and implementing cross-border and cross-disciplinary transition programmes that include establishing transition teams between CAHS and AHS, conducting transition workshops for young patients, and developing digital solutions. The present research protocol outlines the research component of the CAT project. The research focuses on young persons' experiences and everyday lives, their health and well-being during the period where they transfer from CAHS to AHS, and on how they and their parents or guardians and health-care professionals experience this period. The project also explores how some of the research participants experience and reflect upon the initiatives in the CAT transition programmes, including their contact with the transition teams, their participation in the transition workshops, and their skill in piloting CAT's digital tools.

## 2 Background

### 2.1 Transition processes from child- and adolescent health-care services (CAHS) to adult health-care services (AHS)

In Denmark and Germany, every fifth young person lives with a serious or chronic illness or diagnosis and relies on support from their families and health-care professionals (10, 11). When they turn 18 years they generally transfer from CAHS to AHS (12). The transfer in medical care occurs at a time in the young persons' life when many physical, mental, and social changes are taking place, and when they are affected by a multitude of other transition processes (e.g., leaving school) that influence how they define themselves as young adults in society. The concepts of transition and transfer, though closely related, have distinct meanings and implications. Transfer refers to a specific event or series of events in which a young person moves from child or adolescent to adult healthcare services (13). Transitions, on the other hand, are broader processes initiated by critical events, such as the transfer between medical departments, and encompass the resulting changes (14). They involve various stages, milestones, and pivotal moments and can be understood through both the processes they entail and their eventual outcomes (14). Unlike transfer, which is a discrete event, transition includes a longer-term process that involves social and developmental adjustments during this period (15). In essence, transition is an ongoing process of preparation for and adaptation to significant life changes, with transfer representing one component of this broader journey (13, 14).

Practices in transition and transfer from CAHS to AHS vary. Young patients report that they prefer to begin transition preparation early, around 15 or 16 years, to allow adequate time for adaptation and skill-building (16). In Germany, national Transition Guidelines recommend starting preparation at 16 years and emphasize that the transfer should not be rigidly tied to the biological age of 18. Instead, the timing should consider the patient's specific condition and readiness (17). Furthermore, differences exist between somatic and mental healthcare. In somatic health care the mean transfer age ist 18 years (12). The World Health Organization (1) highlights that only three European countries set the transfer age in mental health lower than 18, typically at 16 years. This variation underscores the importance of tailoring transition practices to the individual's developmental, clinical, and psychosocial needs.

Yet, the transfer in medical care generally takes place irrespective of a person's readiness to transition or their developmental stage (12). The review by Hill et al. (18) describes the differences in the service culture between CAHS and AHS, showing that CAHS has a broader focus on adolescents' everyday lives and well-being and that disease management is only one aspect of many. It is more family-oriented in involving the parents or guardians more, and adolescents have less responsibility for managing their medical treatment. The circumstances are different with AHS, where young patients are required to be more autonomous, managing their treatment more independently with less involvement by their parents or guardians. Treatment at AHS is more disease- or diagnosis-focused, because less time is allowed for consultation. As a result, some young patients feel overlooked or even rejected by health-care providers in AHS, feeling that their disease or diagnosis is more in the foreground than they are as individuals (6, 19).

Another difficulty in the broader transition process for many young patients is that frequently there is a gap between the end of the treatment at CAHS and the beginning of the treatment at AHS (4, 5, 12). Young patients, as well as health-care professionals, criticise the lack of communication between CAHS and AHS in the transfer between departments, including the lack of information and preparatory consultations related to the transition (18–20). CAHS professionals are often unsure of the treatment the patient will receive at AHS and will therefore focus mostly on ending their treatment relationship rather than preparing the young patients for transitioning to AHS (19). The lack of free places for admission to AHS is another significant barrier that impedes successful transfers, since some patients never transfer but rather merely end their treatment in CAHS (12). This is especially a concern in mental-health care, where many young patients do not know who to contact and how to reach out for readmission if they need further support.

While patients, parents or guardians and health-care professionals share similar views on the challenges and barriers in the transition process, there are also different views of this. For instance, parents or guardians and health-care professionals share concerns regarding the differences between the structures and foci of CAHS and AHS and their deficits in knowledge and responsibilities, guidance and protocols for the transition process, and the level and type of service provision (18). Indeed, clinicians report worries of possibly failing patients due to their limited flexibility (8). Meanwhile parents and caregivers repeatedly request psycho-education and parent support groups (21), as they feel restricted in their involvement in AHS (22).

#### 2.1.1 Transition readiness

Transition readiness refers to the development of all the necessary skills (e.g., disease-specific knowledge and self-management skills) for a successful transition (23). This definition implies that, besides biological age, there are further criteria that determine the ability of young persons to cope with and manage the transition process successfully. Sawicki and colleagues (24) propose self-management and self-advocacy as two components of transition readiness. Self-management encompasses skills in disease management (e.g., medication, appointments, health insurance) while self-advocacy describes levels of autonomy in handling everyday life beyond the disease.

A low level of transition readiness can have serious consequences, as patients who report not feeling prepared show an increase in psychological distress and are more likely to develop a new disorder during the transition process (25). Lugasi et al. (26) found that adolescents prefer to receive information in various formats about their new providers and treatment methods, as well as the structural differences between CAHS and AHS. Young patients also want to be more involved in planning and decision-making during the transition process (27). For example, they recommend joint visits to the AHS before the transfer takes place, as well as having peer-to-peer mentors who have already experienced the transfer (26). For an optimal transition process, the transition preparation should start well in advance of the transfer between the two departments (21).

Varty and Popejoy (23) identified four categories of factors associated with transition readiness: demographic factors (e.g., age, gender), psychosocial factors (e.g., self-efficacy, social support), transition-related education (e.g., in self-management and disease knowledge, discussions about the transition) and health outcome factors (23). Other studies identify age as an important factor, since, with increasing age, young patients show more transition knowledge and skills (28, 29). Indeed, 50% of transition skills are not fully accomplished until patients are 18 years old or older (24, 29). However, age does not predict levels of treatment adherence (28). Further favourable demographic factors include being of female sex and living in higher-income households, which are associated with better skills in communication abilities, as well as participation in school, household and community activities (24, 28). Female patients also score higher on transition knowledge (24, 28). Furthermore, young patients with physical health problems achieve higher levels of transition readiness compared to young patients with mental or cognitive challenges (24). The latter finding highlights how young patients facing mental or cognitive challenges specifically need support to improve their transition readiness.

#### 2.1.2 Parental roles in transitions from CAHS to AHS

There is only limited research on the perspectives of the parents and guardians of young patients with a serious or chronic health condition, especially regarding how they experience their child's transition and the transfer from CAHS to AHS. Parents are informal, unpaid health-care managers who have a wealth of knowledge that both their child and health-care professionals can draw on. In one of the few studies available in rheumatology, 78.6% of parents said that they feared the transition process (30). Parents often feel unprepared for the shift in responsibility (19, 31), criticising the fact that they are often not as involved in their child's treatment after the latter transfer to AHS (18); they therefore fear missing out on important information about their children's medical health (31). A major concern for parents is trusting a new doctor with the treatment of their child (30), as they fear that the new health-care providers at AHS have only limited knowledge about their child's health condition and individual care needs during the transition process. At the same time, some parents also describe feeling relieved after the transfer to AHS because they no longer have all the responsibility for managing their child's medical treatment (18). Sönmez et al. (32) found that parents often believe that their children are not as ready for transitioning as the young persons' own perceptions of their level of readiness. Hart et al. (33) argue that parents who feel overwhelmed by their sense of responsibility (“role overload”) are more likely to conclude that their child is not transition-ready. This negatively influences the young person's transition process and underlines the importance of taking the parents' transition readiness into account in order to improve the transition process for young adults. A recent study shows that barriers to the parents' readiness for handling their child's transition also arises from a lack of support from health-care providers, who may lack knowledge of when and how to involve the parents during the process (34). Top-down decision-making or uncertainties over how to participate in join consultations can lead to parents distrusting the health-care system or failing to understand what their new role is (34).

#### 2.1.3 Youth transition in rheumatology and mental health care

There is a lack of literature presenting evidence for effective and patient-focused transition preparation (35). Common transition preparation programmes include education about the patient's condition, treatment and overall issues faced by young patients during this time, and the promotion of autonomous behaviour (26). Besides nurturing transition readiness (4, 36), young patients also identify the need to determine the differences between CAHS and AHS as essential before transfering between departments (7). Other studies show that social support is of crucial importance to young patients and their families during the transition process, expressing a need for transition programmes that attend to other issues of concern, and not only the disease or diagnosis and medical treatment (31, 37). For instance, this includes help with social and psychological problems during the transition period (31, 37).

Transition preparation interventions have been found to be effective, resulting in continuity of care, fewer drop-outs and health improvements (9). For instance, these interventions suggest raising the age of transfer to 25 years, developing specialized services for patients from 16 to 24 years (12, 38), or establishing follow-up meetings with a CAHS professional after the transfer to AHS has taken place (18).

The transition process from CAHS to AHS has been widely studied in patients with certain chronic, somatic medical fields (e.g., diabetes). Studies indicate that the number of emergency visits of patients with chronic diseases increases at the age of transfer, while planned admissions decrease post-transition (39). In the CAT study we focus on two medical fields, namely rheumatology and mental health care.

The research focuses on two distinct patient populations—rheumatology and mental health—with the aim of comparing and integrating perspectives across somatic and mental health care. By including rheumatology, a long-term chronic somatic condition, alongside mental health, the study provides a unique opportunity to explore commonalities and differences in transition experiences across these domains, fostering a more holistic understanding of youth transition in healthcare.

A common disease treated in rheumatology is juvenile idiopathic arthritis (JIA). JIA is the most common chronic heterogenous rheumatological disorder that manifests in patients aged less than 16 years and globally three million children and young adults are diagnosed with JIA (40). Most of these young patients are confronted with transfering from CAHS to AHS at a time in their lives when they have only recently been diagnosed and are still learning to live with their chronic disease alongside other puberty-related changes (40). There is limited qualitative research examining how young patients with JIA experience the transfer from CAHS to AHS and how they experience the broader transition process. The few studies available suggest that the challenges are similar to transition processes for patients with other chronic or long-term illnesses (4, 5, 37). Many studies argue that there is a high risk of patients getting lost in the transition process where they transfer from the CAHS paediatric rheumatology department to the AHS rheumatology department. This is demonstrated in a study focusing on patients with JIA, where 53% of the patients had an unsuccessful transfer, i.e., did not meet with their adult rheumatologist or only presented in AHS two years after leaving CAHS (3–5).

Similarly, studies focusing on the transfer from CAHS to AHS in mental health care highlight significant deficits, which results in only 24% of the reviewed adolescents transferring after they have turned eighteen (41). This is of particular concern given that most psychiatric disorders have an average onset at this age. Some studies identify factors that influence adolescents' decisions to avoid mental health-care treatment to include the fear of social stigma and self-stigma, a lack of physical access to the services, concerns about confidentiality, a lack of information and losing psychosocial support (21).

### 2.2 Project: Child to Adult Transitions (CAT)

The transition from CAHS to AHS is at the heart of the cross-border, cross-disciplinary project “*Child to Adult Transitions: Enabling Good Transition Processes From Child To Adult Medical Care” (CAT).* The CAT project runs from January 2023 to December 2025. This study protocol concerns the research to be carried out within the CAT project. However, since the research also includes an investigation of how the participants experience the transition programmes, a brief background to the project is provided in this section (project website: https://forskning.eu/projects/cat-child-to-adult-transitions/).

The project involves nine partners from hospitals and/or research institutions in the northern regions of Germany and the south and southeast regions of Denmark. There are important differences between the two countries involved, especially regarding the financial structures of medical care. In Denmark, all larger hospitals are public, whereas in Germany many of the hospitals are run by private providers (42). Denmark mainly relies on a public insurance scheme, which covers the expenses for primary and secondary health care, and patients are responsible for selecting a general practitioner in their own municipality. Germany, on the other hand, has 96 competing health insurance companies (43), where the patients are free to choose a practitioner irrespective of residence. These structural differences are taken into account in both the project implementation and the research design.

The value of the project is that it exchanges experiences and knowledge for creating cross-border and cross-disciplinary transition programmes that can also provide guidance to other hospitals and disciplines in the future. Combining somatic and mental health care presents another advantage since it allows for comparison across disciplines. This is highly relevant given that up to 20% of young patients in rheumatology also have mental health concerns (44).

The CAT project aims to develop cross-border transition programmes for young patients aged 15–25 years, which support them as they transfer from CAHS to AHS. These programmes will enable closer contact between the patients, parents/guardians, and health-care professionals. Three types of transition programmes are developed: transition teams, transition workshops, and digital solutions.

#### 2.2.1 Transition teams

A central component in developing, implementing and executing the transition programmes will be the formation of transition teams. These teams will operate cross-departmentally within the same hospitals and countries by connecting CAHS to AHS. They will support the patients with guidance, consultations and workshops. The transition team may consist of a doctor, nurse and/or other health care professionals who follow the guiding principles for transition support (45).

#### 2.2.2 Transition workshops

To build the competencies, knowledge and networks among young patients who will transfer from CAHS to AHS, transition workshops will be implemented. The workshops are based on and adapted from the KomPaS workshop manuals (46–48) that are designed to focus on patients with a chronic, somatic illness in Germany. In the CAT study, the workshop material will be adapted to young patients in mental health care, and also made suitable for the Danish context. The KomPaS workshops are quality-assured and undergo a continuous evaluation process; see Ernst and Szczepanski (48) for details. The workshop consists of nine modules, which address different topics, for example, “Finally 18! Transfer to adult healthcare”, “The new doctor”, “Career start”, and 'Social network' (46). The transition workshops facilitate young patients' independence since they learn to take responsibility for their own treatment, and the cross-cultural workshop also facilitates networks with other peers across borders.

#### 2.2.3 Digital health solutions

In Germany, political attention towards implementing digital health technologies has increased recently, but digital infrastructure has not been developed sufficiently to support the implementation accordingly (49). Hence, Danish advances in these technologies can serve as the infrastructure for developing innovative digital solutions supporting young patients on both sides of the border. In the CAT project, Trifork (2024), a company specializing in digital health solutions, aims to define and develop digital tools that support the young patients in their transfer from CAHS to AHS. User-driven innovation plays a central role in this, and the digital solutions are developed in close interaction with adolescents and young adults during co-creation workshops. The digital solutions are designed as long-term solutions and should therefore address the needs and interests of young patients. The solutions could, for example, include a digital app or website, a messaging and/or video-sharing platform, etc. and allow access to information, the tracking of own treatment processes and communicating with health-care professionals.

### 2.3 CAT Study rationale and research questions

This project and its research are novel in several key ways, offering a unique contribution to the field of transition studies: First, the integration of mental health and somatic care sets our study apart. While most research tends to focus on either mental health or somatic conditions in isolation, our work bridges these domains. Second, our longitudinal approach distinguishes this project. We follow patients and parents throughout the transition period, encompassing both pre- and post-transfer phases. Unlike most studies, which predominantly address the pre-transfer or post-transfer period, our research spans the full timeline. This enables us to capture the evolving experiences, challenges, and coping mechanisms, offering a more complete and nuanced perspective on this critical process. Finally, our interdisciplinary perspective combines insights from the medical (symptoms and treatment options), psychological (personal experiences), and anthropological (intersubjective relations and everyday life) fields to explore the multifaceted nature of transitions and medical transfers.

It is expected that conducting research with young patients, their parents or guardians, and health-care professionals will (a) generate knowledge about their transition experiences, (b) enhance understanding of the potential challenges in the transition process, and (c) illuminate the potential for providing more support and attention to young patients' needs. This new knowledge serves to develop and improve the CAT transition programmes.

#### 2.3.1 Main research question

How do young persons experience and reflect upon their transition from child to adult medical care, and how do they experience the CAT transition programmes?

#### 2.3.2 Sub-research questions

What are the similarities and differences in the transfer from CAHS to AHS across the disciplines of rheumatology and mental health?

What are the similarities and differences in the transfer processes from CAHS to AHS between Germany and Denmark (specifically between Schleswig-Holstein and Region Zealand)?

## 3 Methods/design

### 3.1 CAT Study design

**Figure 1:**
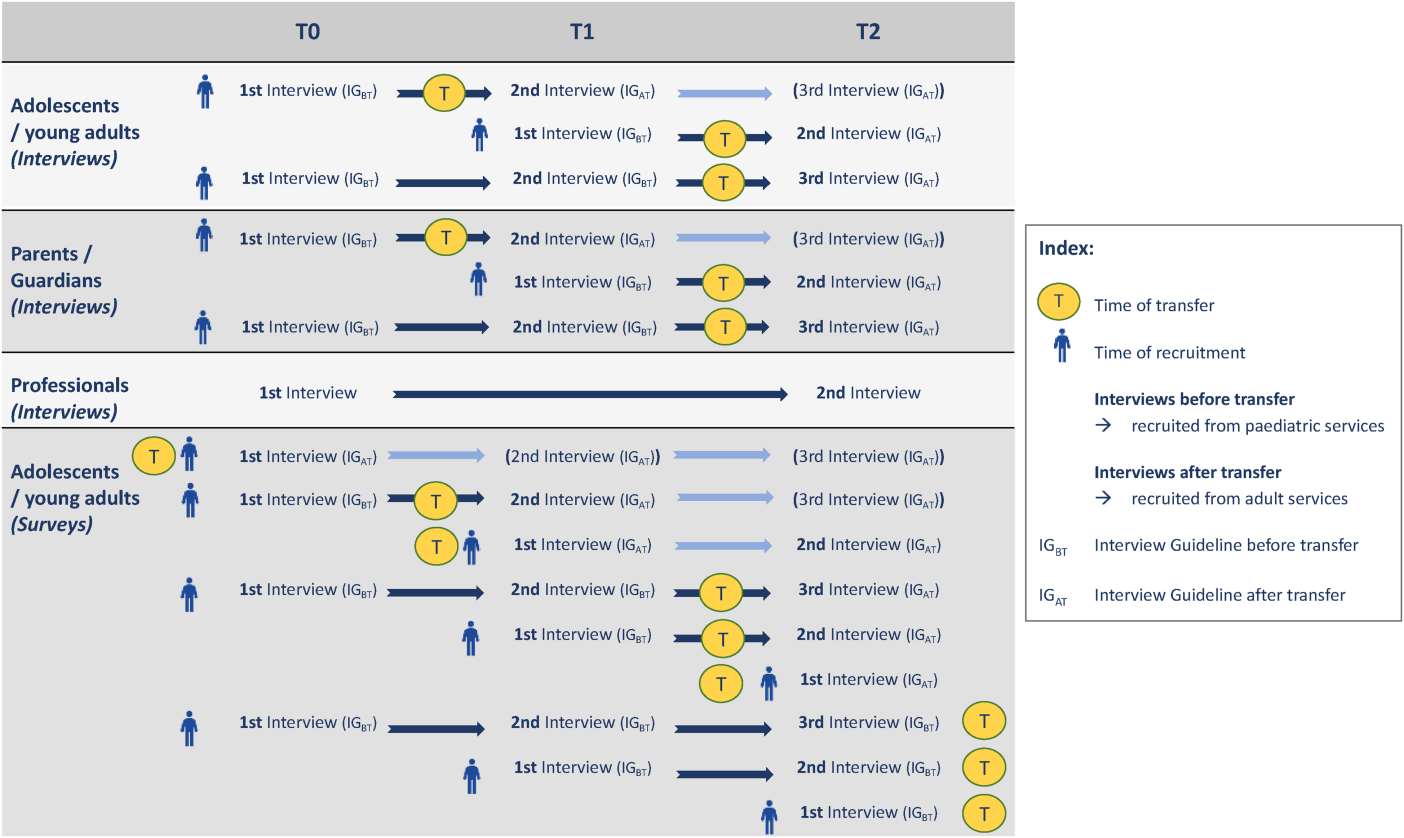
Overview of the CAT research design. Note. T0 = Baseline, T1 = after 6 months, T2 = after 12 months. Parents, guardians and medical professionals will only participate in the qualitative study. Adolescents and young adults will be invited to participate in the qualitative interviews and observations, as well as the quantitative online survey. For interview participants the transfer takes place at some point between T0 and T2, while for the survey the transfer can also take place before or after T2.

The CAT study design is designed to be a longitudinal mixed-methods study (see Figure 1). It will include qualitative research consisting of individual or group semi-structured interviews with young patients, their parents and guardians, and health-care professionals, as well as observations during consultations, transition workshops and digital co-creation workshops. The project will also include a quantitative online survey administered to young patients. The interviews and surveys will take place at three time points, including at baseline (T0), after six months (T1) and after twelve months (T2). The observations during consultations and the digital co-creation workshops will take place at T0, and the observations during the transition workshops at T1.

### 3.2 Study population

#### 3.2.1 Inclusion and exclusion criteria

**Adolescents and young adults** should

(a) Be in treatment^1^ in a rheumatology or mental health-care department in Region Zealand, Denmark, or Schleswig-Holstein, Germany.
(b) They should be between the ages of 15 and 25 years.^2^
(c) For the qualitative interviews, only participants who are likely to transfer from CAHS to AHS between T0 and T2 are included.

**Parents and guardians** of adolescents and young adults are included if they have one or more children that

(a) Are in treatment in a rheumatology or mental health-care department in Region Zealand, Denmark, or Schleswig-Holstein, Germany,
(b) Are between the ages of 15 and 25 years, and
(c) Are likely to transfer from CAHS to AHS between T0 and T2.

Health-care professionals are included if they

(a) Are working in a CAHS or AHS department that is part of the CAT project and
(b) Are in contact with young patients transferring to or from CAHS to AHS.
(c) However, health-care professionals involved in the research design of the CAT study will be excluded.

The exclusion criteria, for all three groups, pertain to proficient language skills.

In the rheumatology department, patients are primarily treated for juvenile idiopathic arthritis (JIA) and other forms of arthritis. In the mental health department, patients receive treatment for a broad range of conditions, including psychosis, depression, anxiety, and autism.

Furthermore, in Denmark, a small group of young persons over 18 years who have transferred from CAHS to AHs in rheumatology and mental health care without receiving transition-related support will be interviewed (*n* = 6–10). These interviews provide baseline insights into the challenges that young adults experience when going through the transition process without any targeted support during the transferring stage and the impact that this has on their everyday lives. The knowledge and topics raised during these initial interviews serve as input for designing the interview guides with the young persons at T0, T1 and T2.

#### 3.2.2 Number of study participants

**Figure 2:**
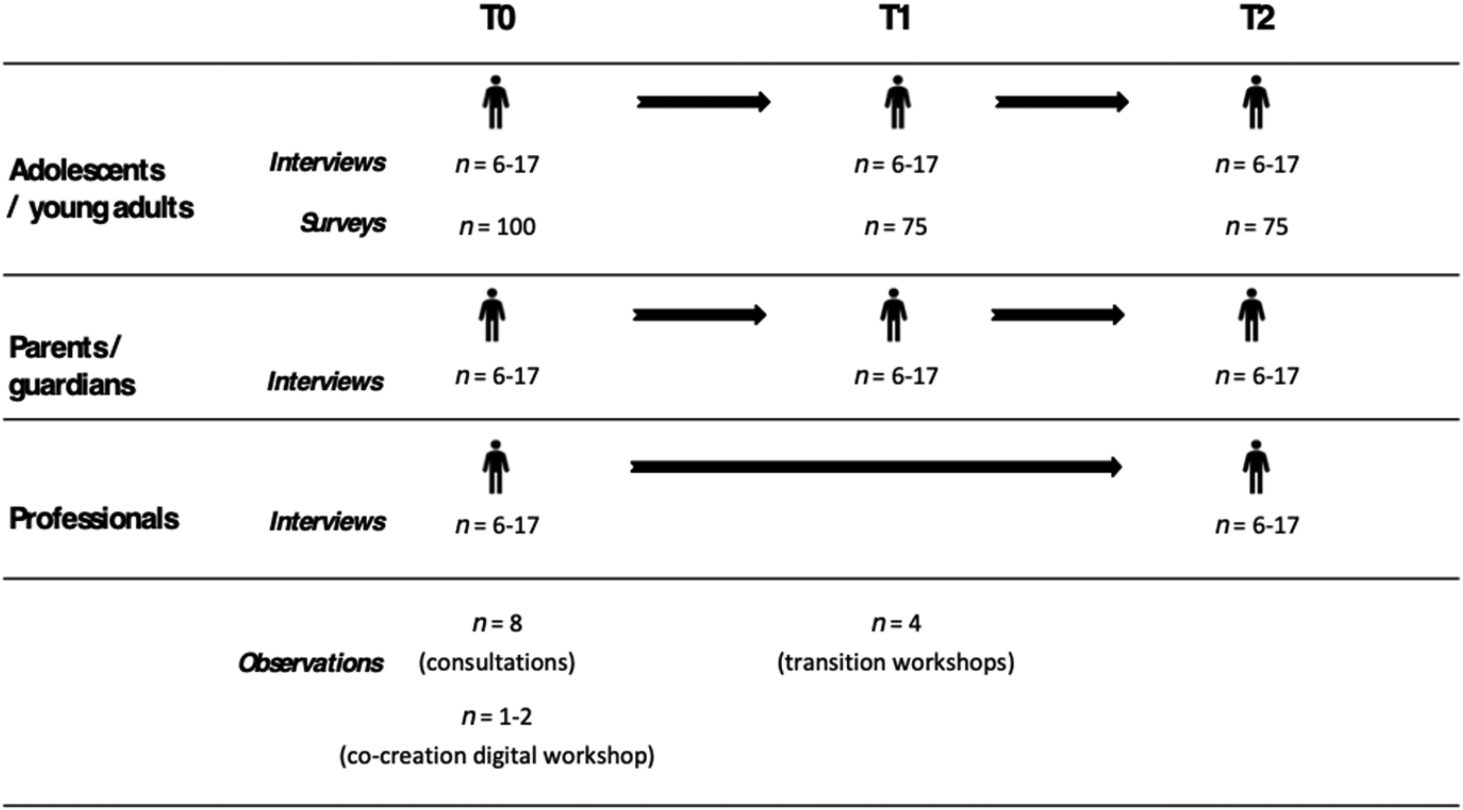
Sample sizes by participant group, research method and time point. Note. Sample sizes for interviews or survey responses are given per country, per discipline. For observations they are given per occasion across countries and disciplines, while one observation at a visit to the health-care department or hospital may include participating in multiple consultations with patients.

See Figure 2 for details of the sample sizes for each participant group (young adults, parents and guardians, health-care professionals), survey method (qualitative interviews and observations, quantitative data) and time points (T0, T1, T2). The sample sizes listed for the qualitative interviews and quantitative surveys of the CAT project are shown for each discipline and country. We aim to recruit balanced samples across sex and age groups.

Recommendations for the number of interviews to be conducted to reach data saturation in qualitative research varies. The focus in qualitative research is not on representation but on gaining crucial in-depth insights into nuances and details of the experiences of the interlocutors that cannot be accounted for in quantitative research. Saturation therefore relates more to exhausting the questions asked (e.g., when information starts to become repetitive) rather than a given sample size (50–52). Based on previous experience and literature, we aim for a sample size of six to seventeen interviews per group, per time-point, per discipline, per country (53, 54). At baseline, this totals interviews with 24–68 adolescents and young adults, 24–68 parents and guardians, and 24–68 health-care professionals in both countries and across disciplines. Interviews will be repeated after six and 12 months. We acknowledge, however, that there can be dropping-out at the follow-ups that may require the number of research participants to be recalculated.

The sample size for this study was determined pragmatically, based on the availability of young patients in the recruiting departments and logistical considerations. A formal power analysis was not conducted, as the study is exploratory. Online surveys will be conducted with 100 young adults at baseline (T0) and 75 participants at follow-up time points (T1 & T2) per discipline per country. This totals 400 survey responses at baseline in both countries and across disciplines, which will be repeated after six and 12 months.

In total, 13–14 observations will be conducted in three settings across countries and disciplines, namely (a) during health-care consultations in the departments and hospitals (*n* = 8 at T0), (b) during the co-creation digital workshops (*n* = 1–2 at T0) and (c) during the transition workshops (*n* = 4 at T1). One observation in the health-care department or hospital may include participating in multiple consultations with patients.

#### 3.2.3 Recruitment strategy

We used a convenience sample from the partner departments. During consultations, health-care professionals will check if adolescents and young patients as well as parents and guardians fulfill the inclusion criteria and invite them to participate in the interviews. Health-care professionals invited to the interviews will be contacted directly by a CAT researcher. In Denmark, recruitment for the online survey will take place through existing online communication channels used in the Danish health-care system (i.e., secure digital mail from health-care providers to patients). In Germany, recruitment for the surveys will take place via health-care professionals, flyers and posters. Observations will be carried out during medical consultations by the CAT researcher, who is granted access by the health-care professionals with the consent of the patient and/or parent or guardian. Observations are also carried out during the digital co-creation workshops and the transition workshops. Here the facilitators of the workshops grant access to the researchers.

A standard incentive is provided to all participants across countries and settings. However, the size of the incentive is adjusted based on local practices to ensure cultural and contextual appropriateness.

The CAT project period runs from January 2023 to December 2025. The recruitment for the CAT study is ongoing.

### 3.3 Data protection and ethics

The study has been approved by the appropriate departments and ethics committees in the relevant countries and regions and will be being conducted in accordance with the ethical standards laid down in the 1964 Declaration of Helsinki, its later amendments or comparable ethical standards, as well international principles for qualitative research (e.g., American Anthropology Association's Principles of Professional Responsibility).

Informed, written consent will be obtained from each research participant prior to the interview, observation, or survey. If participants are under the age of 18 years, informed, written consent will also be obtained from a parent or guardian. Participants are informed about how their data will be used and stored, as well as about their pseudonymised/anonymised participation, and their ability to withdraw from the project at any time without any repercussions will be made clear to them. Information about the study will be provided in a language that is understandable and easy to understand. Contact details are provided so that participants can obtain more information and ask questions.

Ethical guidelines and General Data Protection Regulation (GDPR) regulations will be followed concerning data usage, access to storage and personal information about the participants will be kept in a different secure folder from the interview recordings and can only be accessed by a few of the researchers involved in the study. Data from the qualitative interviews, quantitative surveys and observations will not be linked to each other nor across participant groups. That is to say, parents and guardians can take part in the interviews even if their child does not participate in the interviews or questionnaire survey. Similarly, young patients can choose whether they want to participate in the interviews, the questionnaire survey, both or neither.

### 3.4 Study procedure

For details of the study procedure, see Figure 1 and Table 3. The following sections describe the qualitative research activities (interviews and observations) and the quantitative research activities (survey).

#### 3.4.1 Qualitative research activities: interviews

The interviews (individual or group) will follow an interview guide (see appendix) and are conducted using a semi-structured approach (51, 52, 55, 56). In this context, “semi-structured” means that although key questions form the centre of the interview, participants can also inform and steer the conversation, allowing it to become more relaxed and informal (50). Follow-up questions for each topic will be formulated as a guide to the conversation, which will not, however, necessarily be asked at each interview. Questions are adjusted to the age of the interview participant, and the focus is on establishing rapport and trust with the interview participant (56). All participants will be informed of the possibility of taking a break during the interview, and that they can decide not to answer questions or discuss certain topics if they do not wish to do so. After the interviews, there will be time for a debriefing with the participant if needed. Depending on their preference, the interviews will take place face-to-face either in the participant's home, or at the hospital or in a third location where the participant feels comfortable. Interviews can also be carried out on the phone or in video calls, depending on the participants' preferences. The interviews are expected to last between 45 and 90 min and if desired can be split into two sessions. All interviews will be recorded, and then transcribed.

The interviews will cover topics related to the young persons' or the parents' or guardians' subjective experiences of the transition process with a particular focus on everyday life, challenges and needs with their disease or diagnosis management, and experiences with receiving transition support. Digital habits are also surveyed to gain insights into the digital solutions that can be developed during the CAT project. The same topics and themes are covered in each interview to ensure consistency. However, the focus of the interviews at T1 and T2 is on the changes and developments that have taken place since the previous interview. See Table 1 for an overview of the topics covered in the interviews.

**Table 1:** Overview of the topics of the interviews by participant group*.*

Adolescents/young adults	Parents and guardians	Health-care professionals
Participant's background	Participant's background	Professional's background
		Focus on the young patient in the department
Participant's medical history	(Child's) medical history	
Everyday life as a young person with disease or diagnosis	Everyday life as a parent of a young person with a disease or diagnosis	
Transition preparation	Parent's transition readiness	
The role of parents and guardians	The role of parents and guardians	The role of parents and guardians
First contact with adult services	First contact with adult services	
Experience of their transition process	Experience of their child's transition process	Preparation for the transition to the adult ward
		Perspectives onthe current transferring process from CAHS to AHS including ideasand proposals for changes
Current treatment	Current treatment of the child	
Hopes and goals for the future	Hopes and goals for the future	
Transition programmes (transition teams, workshops, digital habits/solutions)	Transition programmes (transition teams, workshops, digital solutions)	Transition programmes (transition teams, workshops, digital solutions)

At baseline, the interviews with health-care professionals will focus on their expert opinions about the needs of young patients during the transition process and the need for transition-specific programmes. At the follow-up interviews, the health-care professionals will be asked to evaluate the transition programmes that have been developed and implemented in the CAT study.

#### 3.4.2 Qualitative research activities: observations

Field observations are important for acquiring insights into the contexts that shape young patients' experiences with the health-care system (50, 57, 58). See Table 2 for an overview of the topics covered in the observations. In the CAT study, observations will take place during medical consultations at CAHS and AHS. This allows insights to be drawn regarding the conversations and relations between the young patients and health-care professionals, as well as the parents or guardians if they are present. This also allows the observer to take note of the impact of the physical surroundings and non-verbal communication. Being present at both CAHS and AHS makes possible a comparison between the two settings. Furthermore, observations will also be made during the digital co-creation workshops, since these provide insights into how young adults understand digital technologies in relation to their personal health-care management. It also permits insight to be acquired into how they talk about digital technologies among their peers and how they understand and relate to larger debates concerning young people and digital technologies. Similarly, observations are made during the transition workshops, when young patients obtain more information about issues related to the transition and can engage in informal conversations among their peers. Being present during these workshops therefore gives the observer vital information about young patients' experiences of the workshops, the interests they have and questions they pose, as well as the peer-to-peer conversations they engage in.

**Table 2:** Overview of the topics of the qualitative field observations by setting*.*

Medical consultations	Co-creation digital workshops	Transition workshops
Chronological development of the event	Chronological development of the event	Chronological development of the event
Setting	Setting	Setting
Communication (verbal and non−verbal)	Communication (verbal and non−verbal)	Communication (verbal and non−verbal)
Communication (oral and written)	Communication (oral and written)	Communication (oral and written)
Time/tempi	Time/tempi	Time/tempi
Relations	Social interactions	Social interactions
Reactions/responses	Other	Other
Other		

#### 3.4.3 Quantitative research activities: surveys

The quantitative online surveys will be conducted with young participants who are old enough to transfer from CAHS to AHS in the near future or have recently transferred. The surveys will take place at baseline (T0), six months later (T1) and twelve months later (T2). The quantitative online survey will include the following topics (see Table 3 for details): participant's background, transition competence, satisfaction, utilization, needs assessment, functioning and social participation, well-being and health-related quality of life, psychopathology (e.g., depression, anxiety, psychosis), stigma, transition-specific life events (e.g., first job, first romantic relationship), substance use (i.e., non-prescribed drugs and alcohol) and medical compliance. Participant's backgrounds will only be surveyed at baseline, and transition competence will only be surveyed if the young patient has not yet transferred to AHS. All other listed aspects will be surveyed at all three time points (T0, T1, T2). The time to complete the survey at each time point should be approximately thirty minutes.

**Table 3:** Overview of the quantitative instruments and survey time points.

Concept	Instruments	Survey time point			
		Item nrs	T0	T1	T2
(1) First questions	Main treatment department (rheumatology or psychiatry), adolescent or adult department, transition process and participation in the CAT transition programmes	2	✓	✓	✓
(2) Participant's background	Sociodemographic background (e.g., age, sex, education, socioeconomic status), medical history	11	✓		
(3) Transition competence^a^	Transition Competence Scale (TCS) (59)	10	(✓)	(✓)	(✓)
(4) Satisfaction, utilization and needs assessment	YHC-SUN-SF (60)	7	✓	✓	✓
(5) Functioning and social participation (ICF)	WHODAS 2.0 (61)	12	✓	✓	✓
(6) Well-being and health-related quality of life	World Health Organization Well-Being Index (WHO-5) (62)	5	✓	✓	✓
	Self-rated (mental and physical) health (single item SF-12/-36)	2	✓	✓	✓
	EuroHis QOL 8 (quality of life)	8	✓	✓	✓
	Pain (WHOQOL BREF, single item)	1	✓	✓	✓
(7) Psychopathology	Depression (PHQ-9) (63, 64)	9	✓	✓	✓
	Anxiety (GAD-7) (65)	7	✓	✓	✓
	Perception/psychosis (CAPE-9) (66)	9	✓	✓	✓
(8) Stigma	SSCI-8 (67)	8	✓	✓	✓
(9) Transition specific life events (TLE)	Objective and subjective measure of TLE	7	✓	✓	✓
(10) Substance use	Single items from the AUDIT (68) & DUDIT (69)	4	✓	✓	✓
(11) Medical compliance	Two single items querying status quo and fifteen items of the MedUseQ (70)	15	✓	✓	✓

^a^ Only participants who have not yet transferred from the CAHS to AHS will be asked to fill out the TCS.

### 3.5 Analysis

The data material will be analysed using cross-disciplinary analytical approaches from medicine (rheumatology and psychiatry), psychology and anthropology. The qualitative data will be triangulated comparing interviews with observations and contextual data. The analysis and coding of data will be done independently by leading researchers at each site/institution and later gathered in anonymized comparative data analyses across countries and disciplines. The text sequences or sense units are coded, and categories are derived from these contents or assigned to existing categories. The resulting category systems enable structured processing of the content materials. The variety of recruitment strategies will carefully consider them during data analysis and interpretation.

For the quantitative data (online surveys), the transition experiences will be examined descriptively (e.g., information received, age of transfer, satisfaction with transfer). Furthermore, variance-analytical and regressive models will be used to analyse within-person changes at baseline, T1 and T2 as well as between-person differences on measured outcomes (e.g., health outcomes). In addition, moderator and mediator variables will be considered (e.g., transition experiences). Quantitative and qualitative data will be compared across countries and disciplines applying different analytical approaches depending on the theme and analytical focus of the given publication. Within- and between-person models will be used to look at potential differences in outcomes (e.g., transition experiences, YHC-Sun, functioning, psychopathology).

## 4 Discussion

The CAT study is a cross-country, interdisciplinary project that will make use of a mixed methods approach to examine experiences of the transition process, as well as the CAT transition programmes. We expect to gain new insights into the experiences shared by young persons with rheumatological or mental health conditions or diagnoses as they transition from child to adult medical care. By combining different medical fields (rheumatology and mental health), academic disciplines (medicine, psychology, anthropology), countries (Germany, Denmark), person-groups (young patients, parents, health professionals) and methods (interviews, observations, questionnaires), new light can be cast on the medical, psychological and anthropological aspects of the complex nature of the medical transition.

As the CAT study positions itself within the field of transitional care, analytical perspectives on transitional care from medicine, psychology and anthropology will be key to the analysis. This includes approaching the concept of transition not merely as a single point in time, but as a continuous process that affects the person throughout their lives and consists of multiple forms of transition (71). Due to the interdisciplinary nature of the study, the theories that will be applied in the analysis of the data will draw on established theoretical directions within the fields of medicine (rheumatology and mental health), psychology, sociology and anthropology. That is to say, medical research papers will draw on relevant studies within the fields of rheumatology and mental health care and, where possible, also combine the two fields to compare transition experiences and learn from each other.

Adolescents and young adults are the protagonists of the CAT project, and the key analytical perspective will be on their everyday lives as young individuals and everyday life with chronic or serious illnesses or diagnoses (72, 73). This allows closer study of how adolescents and young adults understand and define their personhood in relation to somatic or mental health-care challenges. The comparison of young patients in rheumatology and mental health care is of particular interest, as studies show that young persons with rheumatic diseases are at a greater risk of poor health compared to the reference groups (44). The CAT study is also one of the few studies to examine the transition process by taking a longitudinal approach and following the young persons throughout the broader transition process (before, during and after the transfer at 18 years). As a result, we expect to gain new insights into the everyday lives of young patients and their parents and how they understand and respond to the challenges of the medical transfer. We will further examine how research participants experience and reflect on the CAT transition programmes and will review recommendations for adjustments and improvements. It is important to note, however, that this research will not provide a comprehensive evaluation of the CAT transition programmes, but rather a summary of best practices and recommendations learned throughout the project period.

Since this project concerns adolescents and young adults who live with serious or chronic diseases or diagnosis, close attention is paid to their vulnerability throughout the project. Consequently, many might not be able to participate in all the project interventions, including individual interviews at three time points, digital workshops and transition workshops. In some cases, this might prove overwhelming, and it is therefore expected that some research participants will only join some interventions and not others and that some will drop out altogether. For this reason, the CAT study is not designed as an intervention study but rather focuses on gaining empirical insights into the experiences of the research participants as they engage with the interventions they are capable of being part of. The scientific publications deriving from the CAT project will examine the associations between relevant elements of the transition process and the developed transition programmes. Beyond the scientific domain, the results will feed into guidelines on transitional care that can also be used in other medical disciplines, as well as be prepared as popular publications and other media enabling a broader audience to be reached.

## Footnotes

^1^Being “in treatment” is operationalized as, at baseline (T0) being registered as a patient in the recruiting rheumatology or mental healthcare departments and having treatment that has not been formally concluded, even if the patient is simultaneously receiving care in primary or private settings.

^2^The age range will vary slightly depending on discipline, region and recruitment strategy: Danish rheumatology = 15–25 years, Danish mental health = 17–25 years, German rheumatology (interviews) = 16–25 years, German mental health (interviews) = 17–25 years, and German rheumatology and mental health (online survey) = 16–25 years.
